# Case Report: *De novo* Variants of *KMT2E* Cause O'Donnell-Luria-Rodan Syndrome: Additional Cases and Literature Review

**DOI:** 10.3389/fped.2021.641841

**Published:** 2021-02-18

**Authors:** Yang Li, Lijuan Fan, Rong Luo, Zuozhen Yang, Meng Yuan, Jinxiu Zhang, Jing Gan

**Affiliations:** ^1^Department of Pediatrics, West China Second University Hospital, Sichuan University, Chengdu, China; ^2^Key Laboratory of Obstetrics & Gynecologic and Pediatric Diseases and Birth Defects of the Ministry of Education, Sichuan University, Chengdu, China; ^3^Cipher Gene LLC, Beijing, China

**Keywords:** KMT2E, O'Donnell-Luria-Rodan syndrome, epilepsy, whole-exome sequencing, neurodevelopmental disorder

## Abstract

**Introduction:** O'Donnell-Luria-Rodan syndrome was recently identified as an autosomal dominant systemic disorder caused by variants in *KMT2E*. It is characterized by global developmental delay, some patients also exhibit autism, seizures, hypotonia, and/or feeding difficulties.

**Methods:** Whole-exome sequencing of family trios were performed for two independent children with unexplained recurrent seizures and developmental delay. Both cases were identified as having *de novo* variants in *KMT2E*. We also collected and summarized the clinical data and diagnosed them with O'Donnell-Luria-Rodan syndrome. Structural-prediction programs were used to draw the variants' locations.

**Results:** A 186 G>A synonymous variant [NM_182931.3:exon4: c.186G>A (p.Ala62=)] was found in one family, resulting in alternative splicing acid. A 5417 C>T transition variant [NM_182931.3:exon27: c.5417C>T (p.Pro1806Leu)] was found in another family, resulting in 1806 Pro-to-Leu substitution. Both variants were classified as likely pathogenic according to the ACMG (American College of Medical Genetics and Genomics) guidelines and verified by Sanger sequencing.

**Conclusion:** To date, three studies of O'Donnell-Luria-Rodan syndrome have been reported with heterogeneous clinical manifestations. As a newly recognized inherited systemic disorder, O'Donnell-Luria-Rodan syndrome needs to be paid more attention, especially in gene testing.

## Introduction

*KMT2E* (GenBank: NM_182931.2, MIM: 608444) (1) is also known as *MLL5* and is located in the 2.5-Mb region at chromosome 7q22.1. It was first recognized in leukemia and encoded a functional protein belonging to the lysine N-methyltransferase 2 (KMT2) family (2). The enzymes of the KMT2 family modulate transcription and promote chromatin accessibility by catalyzing the methylation of histone H3 on lysine 4 (H3K4) with their highly conversed SET domain (2, 3). However, SET domains in *KMT2E* were absent from important motifs and were proven to lack intrinsic methyltransferase activity *in vitro*. Unlike a traditional histone methyltransferase, *KMT2E* has been thought to be a transcriptional regulator involved in cell cycle progression (4, 5), genomic stability (6), hematopoiesis, and spermatogenesis (7, 8). However, more studies are needed to explore the detailed molecular mechanisms. In previous research, the *KMT2E* variant was found to contribute to schizophrenia and autism (9). However, in 2019, O'Donnell-Luria et al. reported a case series of *KMT2E* related neurodevelopmental disorder characterized by global developmental delay and named it O'Donnell-Luria-Rodan (ODLURO) syndrome (10).

Because the disease was only recently recognized and only a limited number of cases have been reported, the genotypes and phenotypes have not been fully elucidated. Here, we present two new cases of neurodevelopmental disorder caused by a *KMT2E* variant to enrich the phenotypes and variants of ODLURO syndrome. We also reviewed previously reported cases and attempted to summarize the clinical features and genetic characteristics of *KMT2E* related neurodevelopmental disorder.

## Materials and Methods

### Cases

Informed consent was obtained from the parents and their families. This study was approved by the institutional review board of the West China Second University Hospital. Their clinical manifestations, electroencephalogram (EEG), brain magnetic resonance imaging (MRI), malformations, investigations of other organs, and gene variations were analyzed. We also combined the 43 *KMT2E* variant-related cases reported previously in our analysis. Additional phenotype data and genetic findings for individuals are summarized in Table 1. We attempted to explore the relationship between different phenotypes and genotypes by statistical analysis.

**Table 1:** Clinical information of cases with *KMT2E* mutations in our study.

**Case**	**Sex, age**	**Variant**	**Consequence**	**Epilepsy**	**ID**	**Other features**
O'Donell-Luria et al. case 1	Male, 11 y	c.167delA, (p.Tyr56Serfs*34)	Frameshift	No	Mild	Autism
O'Donell-Luria et al. case 2	Female, 12 y	c.280delA, (p.Thr94Leufs*25)	Frameshift	No	Moderate	Delay;Macrocephaly
O'Donell-Luria et al. case 3	Male, 9 y, 6 m	c.450dupT, (p.Arg151*)	Non-sense	NA	NA	Autism; Delay
O'Donell-Luria et al. case 4	Male, 7 y	c.450dupT, (p.Arg151*)	Non-sense	NA	NA	Autism; Delay
O'Donell-Luria et al. case 5	Male, 6 y	c.450dupT, (p.Arg151*)	Non-sense	NA	NA	Autism; Delay
O'Donell-Luria et al. case 6	Male, 5 y, 9 m	c.556+1G>A	Essential splice site	Yes	NA	Delay
O'Donell-Luria et al. case 7	Male, 12 y, 2 m	c.997delG, (p.Glu333Argfs*32)	frameshift	No	NA	Delay Macrocephaly
O'Donell-Luria et al. case 8	Male, 3 y, 1 m	c.1130+2T>C	Essential splice site	No	Yes	Delay Macrocephaly
O'Donell-Luria et al. case 9	Female, 21 y	c.1239delC (p.Asn414Metfs*4)	frameshift,	yes	moderate	Delay; Macrocephaly
O'Donell-Luria et al. case 10	Female, 8 y	c.1603delC (p.Leu535Tyrfs*15)	Frameshift	NA	NA	Delay;Macrocephaly
O'Donell-Luria et al. case 11	Male, 11 y, 4 m	c.1776_1780delAAAGA, (p.Lys593Argfs*17)	Frameshift	No	Yes	Delay; Macrocephaly
O'Donell-Luria et al. case 12	Female, 3 y, 6 m	c.1776_1780delAAAGA, (p.Lys593Argfs*17)	Frameshift	Yes	Yes	Delay
O'Donell-Luria et al. case 13	Female, 1 y, 10 m	c.1812delG, (p.Ile605Serfs*41)	Frameshift	No	NA	Autism: NA; Delay
O'Donell-Luria et al. case 14	Male, 3 y, 7 m	c.2261delC, (p.Ser754*)	Non-sense	No	Low-normal	Delay
O'Donell-Luria et al. case 15	Male, 4 y, 3 m	c.2452C>T, (p.Arg818*)	Non-sense	No	Mild	Delay
O'Donell-Luria et al. case 16	Male, 8 y	c.2602_2605delACTA, (p.Thr868Hisfs*3)	Frameshift	No	NA	Autism Delay
O'Donell-Luria et al. case 17	Male, 1 y, 7 m	c.2620C>T, (p.Arg874*)	Non-sense	No	NA	Delay
O'Donell-Luria et al. case 18	Female, 3 y, 6 m	c.2936delT, (p.Leu979Trpfs*9)	Frameshift,	No	NA	Delay Macrocephaly
O'Donell-Luria et al. case 19	Male, 4 y, 8 m	c.3070C>T, (p.Gln1024*)	Non-sense	No	NA	Macrocephaly
O'Donell-Luria et al. case 20	Male, 12 y	c.3198delC, (p.Trp1067Glyfs*2)	Frameshift,	No	Mild	Autism Delay: NA Macrocephaly
O'Donell-Luria et al. case 21	Female, 6 y, 5 m	c.3198_3234del, (p.Trp1067Glnfs*2)	Frameshift	No	Mild	Macrocephaly; Delay
O'Donell-Luria et al. case 22	Male, 5 y, 10 m	c.3494_3495delGA, (p.Arg1165Thrfs*3)	Frameshift	No	NA	Macrocephaly; Delay
O'Donell-Luria et al. case 23	Male, NA	c.3527_3530delCAGA, (p.Thr1176Argfs*16)	Frameshift	No	NA	Macrocephaly: NA Delay::NA Autism
O'Donell-Luria et al. case 24	Female, 9 y	c.3554C>G, (p.Ser1185*)	Non-sense	Yes	Mild	Delay
O'Donell-Luria et al. case 25	Male, 6 y	c.3672_3673delTA, (p.Tyr1224*)	Frameshift	No	NA	Delay
O'Donell-Luria et al. case 26	Male, 5 y	c.4397_4398ins19, (p.Pro1467Thrfs*75)	Frameshift, last exon	No	Mild	Macrocephaly; Delay
O'Donell-Luria et al. case 27	Male, 12 y, 10 m	c.4485_4486delTC, (p.Gln1496Lysfs*39)	Frameshift, last exon	No	Mild	Macrocephaly; Delay
O'Donell-Luria et al. case 28	Male, 6 y, 7 m	c.4829dupT, (p.Leu1610Phefs*259)	Frameshift, protein extension	No	Low-normal	Autism:NA Delay:NA Macrocephaly
O'Donell-Luria et al. case 29	Male, 8 y, 8 m	c.4872dupC, (p.Val1625Argfs*244)	Frameshift, protein extension	No	Yes	Macrocephaly; Delay
O'Donell-Luria et al. case 30	Male, 24 y	c.5453_5460delTGGCCCTG(p.Val1818Alafs*48)	Frameshift, protein extension	NO	Moderate	Delay:NA Macrocephaly: relative
O'Donell-Luria et al. case 31	Female, 12 y, 11 m	7:103354482-105407628x1, 2.05 Mb	Microdeletion	No	Moderate	Autism Delay Macrocephaly
O'Donell-Luria et al. case 32	Female, 18 y	7:104678742-104730547x1, 0.052 Mb	Microdeletion	Yes	Moderate	Delay
O'Donell-Luria et al. case 33	Male, 22 y	7:103679146-105547471x1, 1.87 Mb	Microdeletion	Yes	Mild/moderate	Delay
O'Donell-Luria et al. case 34	Male, 7 y	7:104099959-107002808x1, 2.9 Mb	Microdeletion	Yes	Mild	Delay Macrocephaly
O'Donell-Luria et al. case 35	Male, 16 y, 3 m	c.418G>A (p.Val140Ile)	Missense	Yes	NA	Delay Autism Macrocephaly: NA
O'Donell-Luria et al. case 36	Male, 2 y, 5 m	c.850T>C (p.Tyr284His)	Missense	Yes	Severe	Delay Autism: NA
O'Donell-Luria et al. case 37	Female, 2 y, 11 m	c.2720A>T (p.Asp907Val)	Missense	Yes	Severe	Delay Macrocephaly: microcephaly
O'Donell-Luria et al. case 38	Female, 36 y	c.4126C>T (p.Pro1376Ser)	Missense	Yes	Mild	Delay Macrocephaly: microcephaly
Sharawat et al. case 39	Female, 4 y	chr7:g.104751268C > A (p.Pro1341Thr >C)	Missense	No	Mild	Delay, subtle facial dysmorphism, central hypotonia, intermittent choreoathetoid, oculogyric movements, microcephaly
Sharawat et al. case 40	Male, 5 y	chr7:g.104747968G > C (p.Asp1022His)	Missense	Yes	Mild	Delay, autistic, microcephaly, subtle facial dysmorphism
Conforti et al. case 41	Male, 54 y	p.Asn183LysfsTer33 (c.549del)	Deletion	Yes	Mild	Arterial hypertension, global cognitive, deficiency, impaired memory and executive domain, along with apathy and depressive symptoms
Conforti et al. case 42	Female, 24 y	p.Asn183LysfsTer33 (c.549del)	Deletion	Yes	Mild	Macrocrania, amnesic, attentive, visuospatial abilities disorders, and depressive symptoms
Conforti et al. case 43	Male, 19 y	p.Asn183LysfsTer33 (c.549del)	Deletion	Yes	Severe	dysmorphic craniofacial features (large forehead, telecanthus, periorbital fullness, and maxillary protrusion), bilateral congenital clubfoot, spinal scoliosis, cryptorchidism, cerebellar ataxia
Our patient 1: 44	Female, 1	c.5417C>T p.Pro1806Leu	Missense	Focal or generalized tonic-clonic seizures, drug resistance	Profound	Global development delay, Microcephaly, dystonia, cafe au lait spot
Our patient 2: 45	Male, 4	c.186G>A, p.Ala62=	Essential splice site	Generalized tonic-clonic seizures	Mild	Global development delay, autism

*ID, intellectual disability; NA, not available*.

### Whole-Exome Sequencing and Bioinformatics

Peripheral blood samples were collected from the probands and their families. Genomic DNA was extracted. A total amount of 1.0 μg genomic DNA per sample was used as input material for the DNA sample preparation. Sequencing libraries were generated using xGen Exome Research Panel probes (IDT, USA) following the manufacturer's recommendations. Sequencing was performed on the Illumina Novaseq 6000 platform.

Burrows-Wheeler Aligner (BWA) (11) was utilized to map the paired-end clean reads to the human reference genome (hg19). GATK (12) was utilized for the recalibration base quality score, SNP, and short indel calling. The variations were annotated using ANNOVAR (13). Variants were picked up in exonic and splicing regions with a minor allele frequency of ≤0.005 in the SNP database (ExAC_EAS, ExAC_ALL, 1000Genomes, gnomAD). The identified variants were both *de novo* variants that were absent in the parents. These were further validated by Sanger sequencing. The ACMG guideline was used to judge the degree of pathogenicity of both variants to evaluate their pathogenicity.

To understand the biophysical consequences of KMT2E protein sequence changes, structural-prediction programs (HMMER, PHYRE2, InterProScan, and NetPhos) were applied to evaluate the presence of protein domains and major secondary structure elements (e.g., helices, strands, loops, disorder, and post-translational modification sites).

## Results

### Case Report

Case 1 was a full-term, 5-month-old test-tube girl with uncomplicated prenatal and neonatal courses. Her other family members, including her twin sister, were all healthy. This girl had seizure onset at 4 months, with focal to generalized tonic-clonic seizures. One small café au lait spot was found on her trunk and hypotonia of the extremities. She had microcephaly with a head circumference of only 38 cm (OFC <-3 SD). She also presented specific facial features, including a slightly large forehead, prominent cheeks, and long eyelashes (Supplementary Figure 1). However, investigations of other organs (heart, eye, liver, kidney, etc.) were negative. She exhibited a failure to thrive, and her development was delayed and stagnant. Upon admission at 5 months of age, she could not lift her neck or roll over and had a poor reaction to human faces, lights, and sound. The EEG showed multifocal spike waves, sharp waves, spike-slow wave complexes, and slow waves in the left posterior temporal region, while the brain MRI was normal (Supplementary Figure 2, 3). Both blood and urine metabolic screening were normal. The auditory brainstem response and visual evoked potential tests were negative. Her seizures were not effectively controlled even though different combinations of anti-epileptic drugs had been administered, often lasting for 10 min to 1 h for every episode. Oxcarbazepine (40 mg/kg.d), levetiracetam (40 mg/kg.d), valproic acid (30 mg/kg.d), topiramate (8 mg/kg.d), clonazepam (1 mg bid), and a ketogenic diet were tried. The patient is currently taking valproic acid (30 mg/kg.d), levetiracetam (40 mg/kg.d), clonazepam (1 mg bid), and a ketogenic diet. At her latest follow-up assessment at 13 months of age, the seizures were not controlled, but the frequency had decreased to 1–5 episodes per month. Repeated EEG monitoring showed improvement in the epileptiform discharge with moderate and low-amplitude sharp and slow wave discharge, obvious in the occipital region. Her occipitofrontal head circumference (OFC) was only 40 cm (OFC <-3 SD). She had profound global developmental delay, with a Gesell Developmental Scale score of 25.

Case 2 was a 5-year-old boy whose birth was uneventful. He showed global developmental delay with mild autism. He experienced two episodes of febrile seizure at 2 years old when EEG showed slow background waves. On admission, he suffered frequent generalized tonic-clonic seizures that lasted for 30 min, which fulfilled the diagnostic criteria of convulsive status epilepticus. His occipitofrontal head circumference was 50 cm. When he was 3 years old, he could walk without support, speak simple words, and follow simple instructions. Additionally, he had difficulties with social interaction and communication without any tests for intellectual disability or autism spectrum disorder before admission. On the day of admission, he was in a coma with a Glasgow Coma Scale score of nine. He had no neck stiffness, but the Babinski sign was positive. No malformations of other organs were identified. Cytological and biochemical examinations of the cerebrospinal fluid were normal, and etiological tests including herpes simplex virus, cytomegalovirus, Epstein-Barr virus, Japanese encephalitis virus, coxsackievirus, mycoplasma pneumoniae, tubercle bacillus, and fungal culture were all negative. In addition, autoimmune encephalitis and metabolic diseases were excluded by antibody and metabolic screening. The EEG showed sharp spikes in the frontal, temporal, and parietal lobes with a slow background (Supplementary Figure 4). Brain MRI showed cerebellar atrophy, as well as broadening of the lateral and third ventricles (Supplementary Figure 5). He was treated with levetiracetam (50 mg/kg/d), valproic acid (40 mg/kg/d), and clonazepam (2 mg bid). After ventilator weaning and withdrawal of sedatives, the boy still had paroxysmal hypertonia of the extremities. Finally, he was seizure-free 1 month later on a combination of levetiracetam (50 mg/kg/d), valproic acid (40 mg/kg/d), and clonazepam (1 mg bid). By the last follow-up, he had been seizure-free for 6 months. Repeated EEG monitoring showed a generalized low-amplitude wave with an absent sleeping spindle. His Wechsler Intelligence Scale score was 55. A trio whole-exome sequencing test was performed on both patients.

### Variations and Interpretation

**Figure 1:**
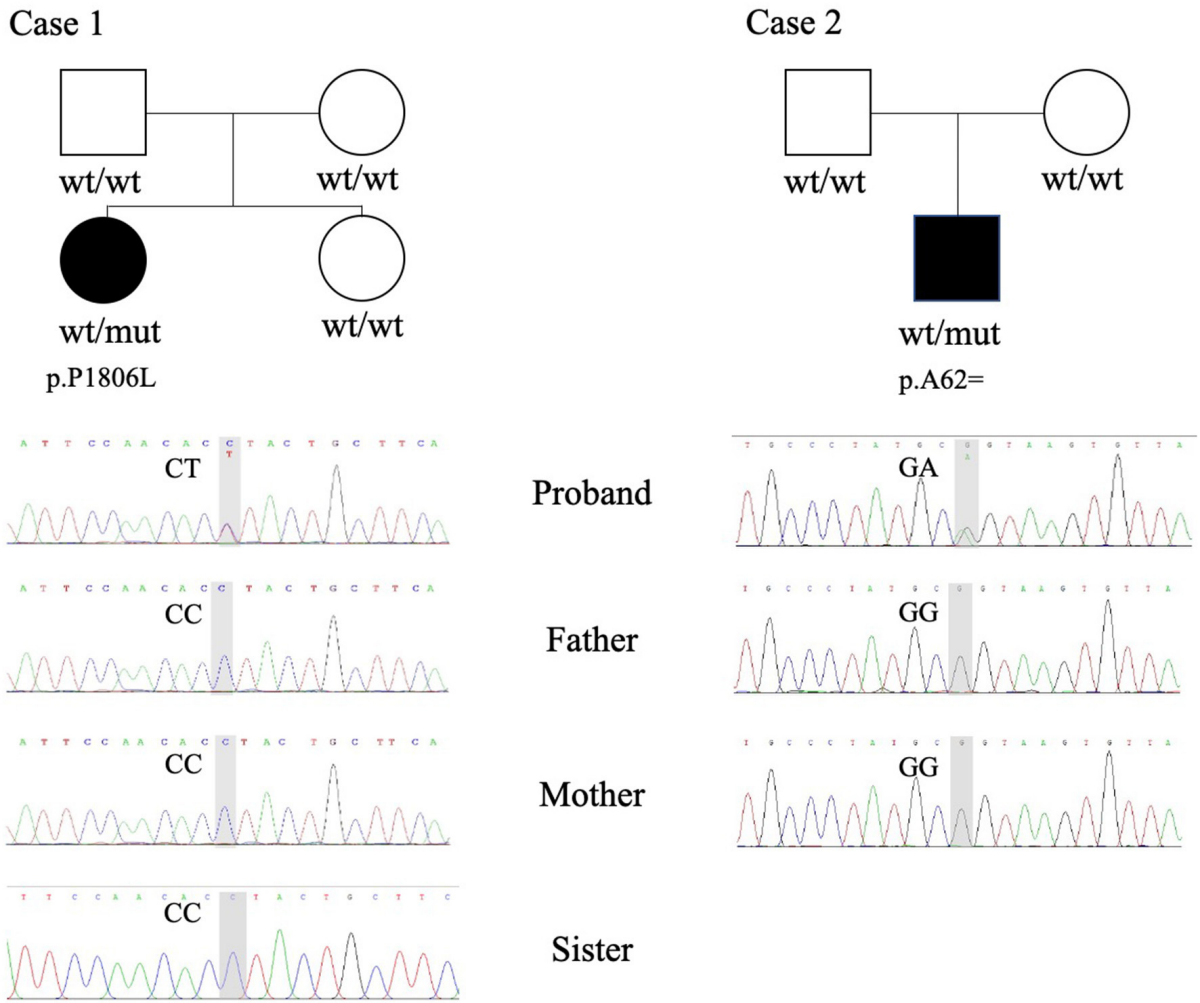
Whole-exome sequencing (WES) and Sanger sequencing revealed heterozygous variants in case 1 (p.P1806L) and case 2 (p.A62=). wt, wildtype; mut, mutation.

**Figure 2:**
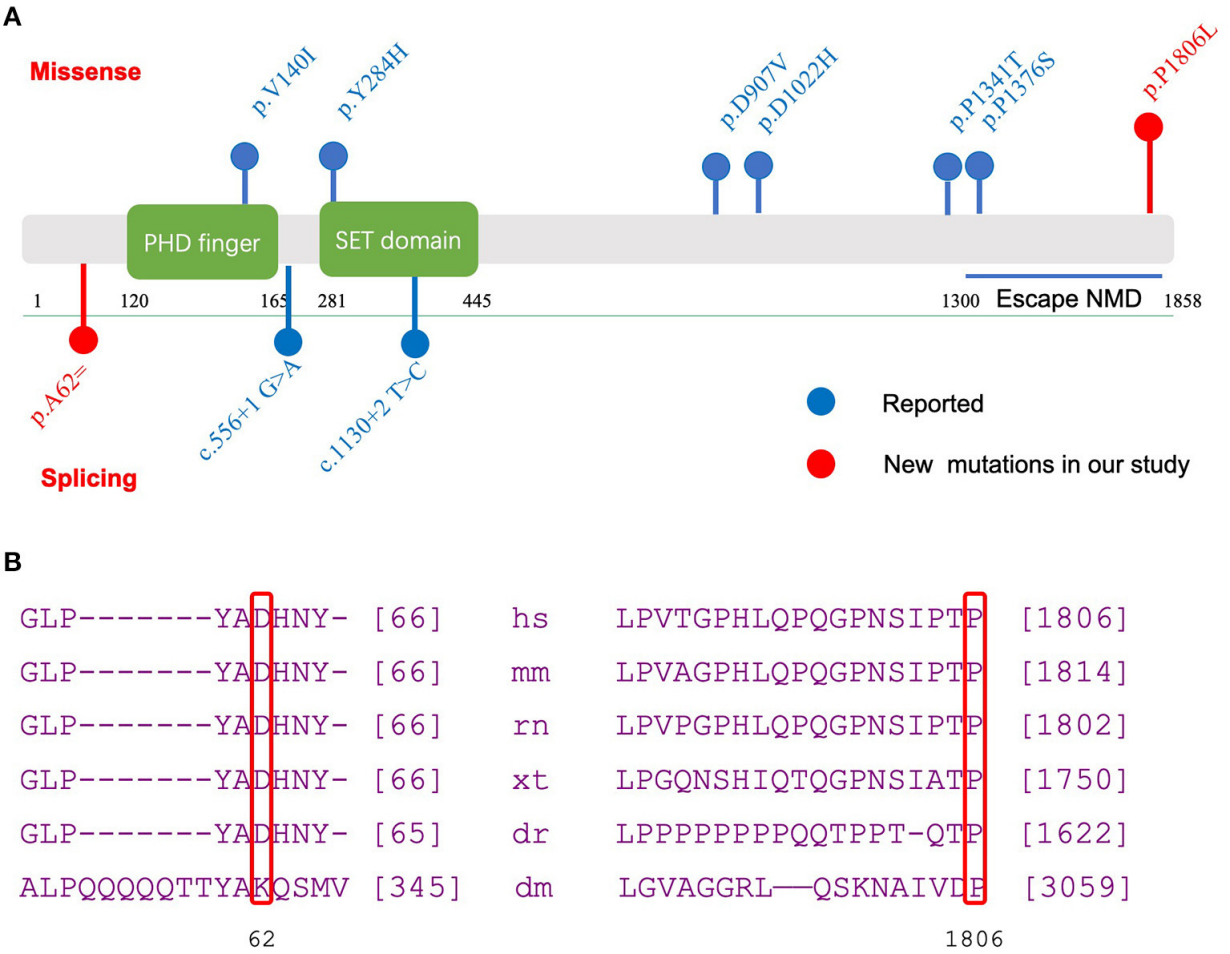
Functional domains of *KMT2E* and evolutionary conservation analysis of amino acid residue. **(A)** Functional domains and regions in *KMT2E* gene as indicated. Red dots: new mutations in our study; Blue dots: reported mutations; Above bar: Missense mutation; below bar: splicing mutation. **(B)** Evolutionary conservation of amino acid residue at position 62 and 1806 in the *KMT2E* gene among species.

Two *de novo* variants in the *KMT2E* gene were identified: a 186 G>A synonymous variant (NM_182931.3:exon4: c.186G>A (p.Ala62=) resulting in alternative splicing acid and a 5417 C>T transition variant [NM_182931.3:exon27: c.5417C>T (p.Pro1806Leu)] resulting in 1806 Pro-to-Leu substitution. Sanger sequencing confirmed the variants in their families (Figure 1). The variant c.5417 C>T results in protein changes from proline to leucine (p.Pro1806Leu) and was predicted to be deleterious by Polyphen2, Mutation taster, and SIFT. As a synonymous variant, the 186 G>A variant was evaluated by “Ada” and “RF” scores from InterVar software (0.9984 and 0.832, respectively, Table 2; threshold: 0.6; higher score means greater probability altering the splicing). The effect of the 186 G>A variant was predicted to be splicing donor site loss, probably resulting in an abnormal isoform (Figure 2A).

**Table 2:** The analysis of pathogenicity of the variants in *KMT2E*.

**Case**	**Sex, age**	**Gene**	**Mutation**	**Inheritance**	**MAF**			**SIFT**	**Polyphen2**	**Mutation taster**	**dbscSNV ADA**	**dbscSNV RF**
					**ExAc**	**gnomAD**	**1000 genome**					
1	Female, 1	*KMT2E*	c.5417C>T	*De novo*	NE	0.00001469	NE	D	D	D	-	-
			p.Pro1806Leu									
2	Male, 4	*KMT2E*	c.186G>A	*De novo*	NE	NE	NE	-	-	-	0.9984	0.832
			p.Ala62=									

*Transcript: NM_182931.3; MAF, minor allele frequency; NE, not exist*.

Further conservation analysis showed that the locus was highly conserved (Figure 2B). The 5417 C>T transition variant was reported once in the gnomAD database. The 186 G>A variant was not found in the public databases (gnomAD, Exome Aggregation Consortium, or 1000 Genomes; Table 2). These two variants were classified as likely pathogenic according to the ACMG guideline (Table 3). Other pathogenic variants of genes known to be associated with epilepsy or intellectual disability were not found in the two probands.

**Table 3:** Pathogenicity classification referred to ACMG guideline.

**Proband**	**Variants**	**Evidence**		**Category**
		**Strong**	**Supporting**	
Case 1	c.5417C>T, p.Pro1806Leu	PS2	PM2_supporting, PP3	Likely pathogenic
Case 2	c.186G>A, p.Ala62=	PS2	PM2_supporting, PP3	Likely pathogenic

We combined all genetic variants and phenotypes from the published cases [43 cases: 38 individuals in 36 families from O'Donell-Luria et al. (10); two individuals in two families from Sharawat et al. (14); three individuals in one family from Conforti et al. (15)] and two cases from two families in our study (Table 1) to explore the relationship between different phenotypes and variants. For each phenotype, we defined the overall incidence of ODLURO syndrome as background and compared each specific phenotype incidence rate in the sub-variant type group to the overall incidence. We found that the incidence rates of ID (intellectual disability) (100 vs. 86.7%, *p* < 0.001), epilepsy (85.7 vs. 41.5%, *p* < 0.001), and microcephaly (66.7 vs. 9.3%, *p* < 0.001) in the missense group were significantly higher than their overall incidence (Table 4). However, no macrocephaly (0 vs. 46.5%, *p* < 0.001) was observed in the missense group (Table 4).

**Table 4:** Phenotype related to variant type.

		**Truncating variants**			**Missense**		
**Phenotype**	**Overall**	**Truncating variants**	***P-*value**	**Trend**	**Missense**	***P*-value**	**Trend**
ID	86.7% (26/30)	80% (16/20)	0.2793	NA	100% (6/6)	0.0004816	↑^***^
Autism	27.5% (11/40)	25.8% (8/31)	0.9109	NA	40% (2/5)	0.08549	NA
Epilepsy	41.5% (17/41)	26.7% (8/30)	0.03955	↓^*^	85.7% (6/7)	2.17E-10	↑^***^
Macrocephaly	46.5% (20/43)	54.5% (18/33)	0.3222	NA	0% (0/6)	2.61E-14	↓^***^
Microcephaly	9.3% (4/43)	0% (0/33)	0.005316	↓^**^	66.7% (4/6)	1.01E-15	↑^***^

* *p < 0.05*;

** *p < 0.01*;

*** *p < 0.001; ↑ occur possibility up; ↓ occur possibility down*.

## Discussion

ODLURO syndrome is a recently reported genetic disease caused by heterozygous variants in the *KMT2E* (the lysine N-methyltransferase 2E) gene (10). To date, 43 cases of ODLURO syndrome have been reported. The clinical characteristics are developmental delay, but macrosomia, cerebellar ataxia, leukoencephalopathy, hypotonia, speech difficulties, memory dysfunction, autism, and other behavioral concerns such as attention-deficit/hyperactivity disorder and depression can also occur (10, 14–17). Many individuals with hypotonia were reported to have feeding difficulties, including reflux, vomiting, or bowel motility issues (10). Patients may have dysmorphic features including a large forehead, full cheeks, prominent nasolabial folds, deep-set eyes with down-slanting palpebral fissures, macrocephaly, bilateral congenital clubfoot, spinal scoliosis, and cryptorchidism (10).

This study described the clinical manifestations of two Chinese patients with ODLURO syndrome from two unrelated families. The cases we reported share the characteristics of other affected patients. Both patients had epilepsy. Case 1 presented with dystonia, profound global developmental delay, and drug-resistant focal seizures or generalized tonic-clonic seizures. Case 1 also had microcephaly which is not a common feature of ODLURO syndrome (4 out of 43 according to reported cases). Case 2 presented with global developmental delay, autism, and generalized tonic-clonic seizures. Of the 43 cases of *KMT2E* variants with neuropathic disorder reported, only 12 had epilepsy, 5 of which were treatment-resistant epilepsy. The main forms of seizures were tonic seizures, clonic seizures, focal or generalized tonic-clonic seizures, myoclonic seizures, epileptic spasms, and absence seizures. There seem to be phenotypic differences between individuals with protein-truncating and missense variants in *KMT2E*. Patients carrying the missense variant might present with a more severe phenotype and are more likely to have seizures than patients with the truncating variant (10, 15). O'Donell-Luria et al. (10) reported that only 5 of 30 patients with the KMT2E protein-truncating variant had epilepsy, of whom only one was treatment-resistant, while all four patients with missense variants had epilepsy, of whom three were treatment-resistant. In addition, patients with a missense variant presented with more severe intellectual disability, and half had microcephaly. The reason is not yet understood but might result from the pathogenic mechanism of haploinsufficiency vs. gain-of-function or dominant-negative effects. More research is required to elucidate this.

The MRI abnormalities of ODLURO syndrome were varied and unspecific. They included thinning or partial agenesis of the corpus callosum, widening of the subarachnoid space, ventricle enlargement, various cysts, increased white matter signal, a hyperintense signal in the basal ganglia, decreased volume, delayed myelination, small areas of heterotopia, cerebellar dysplasia, and Chiari I malformation. Among them, thinning of the corpus callosum may be common and was seen in 7 of 41 patients (although MRI data were limited) (10, 15). In our study, case 1 had normal MRI findings, while the MRI of case 2 demonstrated cerebellar atrophy and broadening of the lateral and third ventricles.

So far, most of the *KMT2E* related ODLURO syndrome cases reported had a *de novo* variant (10). These variants are distributed throughout the gene with no hotspot variant. Most reported pathogenic variants were frameshift and non-sense, and only six missense and two essential splice sites were reported previously (10, 14, 15). Synonymous variants are generally considered non-pathogenic and are not expected to change the function of the protein. However, recent studies have shown that synonymous changes in codon usage may alter the efficiency and speed of translation, ultimately affecting protein folding (18, 19). “Ada” and “RF” scores have been widely used for evaluating potential splicing variants predicted by dbscSNV (13). Both are ensemble scores, which are derived from the outputs of several machine learning algorithms. The score is scaled from 0 to 1, with higher values indicating a greater probability that the variant will alter the splicing of the gene. Moreover, 0.6 was regarded as a threshold value for dichotomous effects. In our study, the Ada and RF scores were 0.9984 and 0.832, respectively. Thus, these scores demonstrate a highly likely splicing result for *KMT2E*.

Our study reported two *KMT2E* variants from two children with *de novo* variants and evaluated the impact of this variant on protein structure. The variant c.186G>A [p.Ala62=] was predicted to influence splicing. The effect of the 186 G>A variant was predicted to be splicing donor site loss, which probably results in an abnormal isoform. The missense variant (c.5417C>T, p.Pro1806Leu) falls in the terminal exon of the gene. Thus, it potentially escapes non-sense-mediated decay and extends the open reading frame. It is predicted to be functionally damaging by several bioinformatics software programs. An evolutionary analysis showed a conserved result at 62 and 1806 sites among multiple species (Figure 2B), indicating a crucial role in gene function. In summary, the variants c.5417C>T [p.Pro1806Leu] and c.186G>A [p.Ala62=] in *KMT2E* can be considered pathogenic and the cause of ODLURO syndrome in these patients.

## Conclusion

Our study adds two new patients to the ODLURO syndrome case series that were genetically, clinically, and radiologically evaluated. Two novel variants, c.5417C>T [p.Pro1806Leu] and c.186G>A [p.Ala62=] were reported, both of which were *de novo* variations. Individuals with missense variants may have a more severe phenotype than individuals with truncating variants. The pathological mechanism of *KMT2E* variants in neurological diseases and the relationship between different genotypes and phenotypes have not been entirely established. Our study adds to the limited information that has been published on ODLURO syndrome and promotes further research.

## Data Availability Statement

The datasets presented in this study can be found in online repositories. The names of the repository/repositories and accession number(s) can be found at: NCBI BioProject, accession no: PRJNA690597.

## Ethics Statement

The studies involving human participants were reviewed and approved by West China Second University Hospital, Sichuan University. Written informed consent to participate in this study was provided by the participants' legal guardian/next of kin. Written informed consent was obtained from the minor(s)' legal guardian/next of kin for the publication of any potentially identifiable images or data included in this article.

## Author Contributions

YL: conceptualization, methodology, and data mining. LF, MY, and JZ: writing (original draft preparation). RL: writing (original draft preparation) and supervision. ZY: software, data mining, and investigation. JG: supervision and writing (reviewing and editing). All authors contributed to the article and approved the submitted version.

## Conflict of Interest

ZY was employed by Cipher Gene LLC. The remaining authors declare that the research was conducted in the absence of any commercial or financial relationships that could be construed as a potential conflict of interest.

## References

[1] Yun H Damm F Yap D Schwarzer A Chaturvedi A Jyotsana N. Impact of MLL5 expression on decitabine efficacy and DNA methylation in acute myeloid leukemia. Haematologica. (2014) 99:1456–64. 10.3324/haematol.2013.101386 24895338 PMC4562534

[2] Zhang X Novera W Zhang Y Deng LW. MLL5 (KMT2E): structure, function, clinical relevance. Cell Mol Life Sci. (2017) 74:2333–44. 10.1007/s00018-017-2470-8 28188343 PMC11107642

[3] Park K Kim JA Kim J. Transcriptional regulation by the KMT2 histone H3K4 methyltransferases. Biochim Biophys Acta Gene Regul Mech. (2020) 1863:194545. 10.1016/j.bbagrm.2020.194545 32194213

[4] Ali M Rincon-Arano H Zhao W Rothbart SB Tong Q Parkhurst SM. Molecular basis for chromatin binding and regulation of MLL5. Proc Natl Acad Sci USA. (2013) 110:11296–301. 10.1073/pnas.1310156110 23798402 PMC3710826

[5] Deng L-W Chiu I Strominger JL. MLL 5 protein forms intranuclear foci, and overexpression inhibits cell cycle progression. Proc Natl Acad Sci USA. (2004) 101:757–62. 10.1073/pnas.2036345100 14718661 PMC321754

[6] Liu J Cheng F Deng LW. MLL5 maintains genomic integrity by regulating the stability of the chromosomal passenger complex through a functional interaction with Borealin. J Cell Sci. (2012) 125(Pt 19):4676–85. 10.1242/jcs.110411 22797924 PMC3500868

[7] Heuser M Yap DB Leung M de Algara TR Tafech A McKinney S. Loss of MLL5 results in pleiotropic hematopoietic defects, reduced neutrophil immune function, and extreme sensitivity to DNA demethylation. Blood. (2009) 113:1432–43. 10.1182/blood-2008-06-162263 18854576

[8] Yap DB Walker DC Prentice LM McKinney S Turashvili G Mooslehner-Allen K. Mll5 is required for normal spermatogenesis. PLoS ONE. (2011) 6:e27127. 10.1371/journal.pone.0027127 22069496 PMC3206077

[9] Shen E Shulha H Weng Z Akbarian S. Regulation of histone H3K4 methylation in brain development and disease. Philos Trans R Soc Lond B Biol Sci. (2014) 369:1652. 10.1098/rstb.2013.0514 25135975 PMC4142035

[10] O'Donnell-Luria AH Pais LS Faundes V Wood JC Sveden A Luria V. Heterozygous variants in KMT2E cause a spectrum of neurodevelopmental disorders and epilepsy. Am J Hum Genet. (2019) 104:1210–22. 10.1016/j.ajhg.2019.03.021 31079897 PMC6556837

[11] Abuin JM Pichel JC Pena TF Amigo J. BigBWA: approaching the Burrows-Wheeler aligner to Big Data technologies. Bioinformatics. (2015) 31:4003–5. 10.1093/bioinformatics/btv506 26323715

[12] McKenna A Hanna M Banks E Sivachenko A Cibulskis K Kernytsky A. The genome analysis toolkit: a MapReduce framework for analyzing next-generation DNA sequencing data. Genome Res. (2010) 20:1297–303. 10.1101/gr.107524.110 20644199 PMC2928508

[13] Li Q Wang K. InterVar: Clinical interpretation of genetic variants by the 2015 ACMG-AMP guidelines. Am J Hum Genet. (2017) 100:267–80. 10.1016/j.ajhg.2017.01.004 28132688 PMC5294755

[14] Sharawat IK Panda PK Dawman L. Clinical characteristics and genotype-phenotype correlation in children with KMT2E gene-related neurodevelopmental disorders: report of two new cases and review of published literature. Neuropediatrics. (2020). 10.1055/s-0040-1715629 33111303

[15] Conforti R Iovine S Santangelo G Capasso R Cirillo M Fratta M. ODLURO syndrome: personal experience and review of the literature. Radiol Med. (2020). 10.1007/s11547-020-01255-2 32691224

[16] Dong S Walker MF Carriero NJ DiCola M Willsey AJ Ye AY. *De novo* insertions and deletions of predominantly paternal origin are associated with autism spectrum disorder. Cell Rep. (2014) 9:16–23. 10.1016/j.celrep.2014.08.068 25284784 PMC4194132

[17] Abdelhalim AN Alberico RA Barczykowski AL Duffner PK. Patterns of magnetic resonance imaging abnormalities in symptomatic patients with Krabbe disease correspond to phenotype. Pediatr Neurol. (2014) 50:127–34. 10.1016/j.pediatrneurol.2013.10.001 24262341

[18] Kimchi-Sarfaty C Oh JM Kim IW Sauna ZE Calcagno AM Ambudkar SV. A “silent” polymorphism in the MDR1 gene changes substrate specificity. Science. (2007) 315:525–8. 10.1126/science.1135308 17185560

[19] Edwards NC Hing ZA Perry A Blaisdell A Kopelman DB Fathke R. Characterization of coding synonymous and non-synonymous variants in ADAMTS13 using *ex vivo* and *in silico* approaches. PLoS ONE. (2012) 7:e38864. 10.1371/journal.pone.0038864 22768050 PMC3387200

