# Supplementary material for: Case Report: De novo Variants of KMT2E Cause O'Donnell-Luria-Rodan Syndrome: Additional Cases and Literature Review
Source: Front Pediatr. 2021 Feb 18;9:641841. doi: 10.3389/fped.2021.641841 (PMC7935518; doi:10.3389/fped.2021.641841)
Supplement: Supplementary file 1 [file Image_1.pdf]

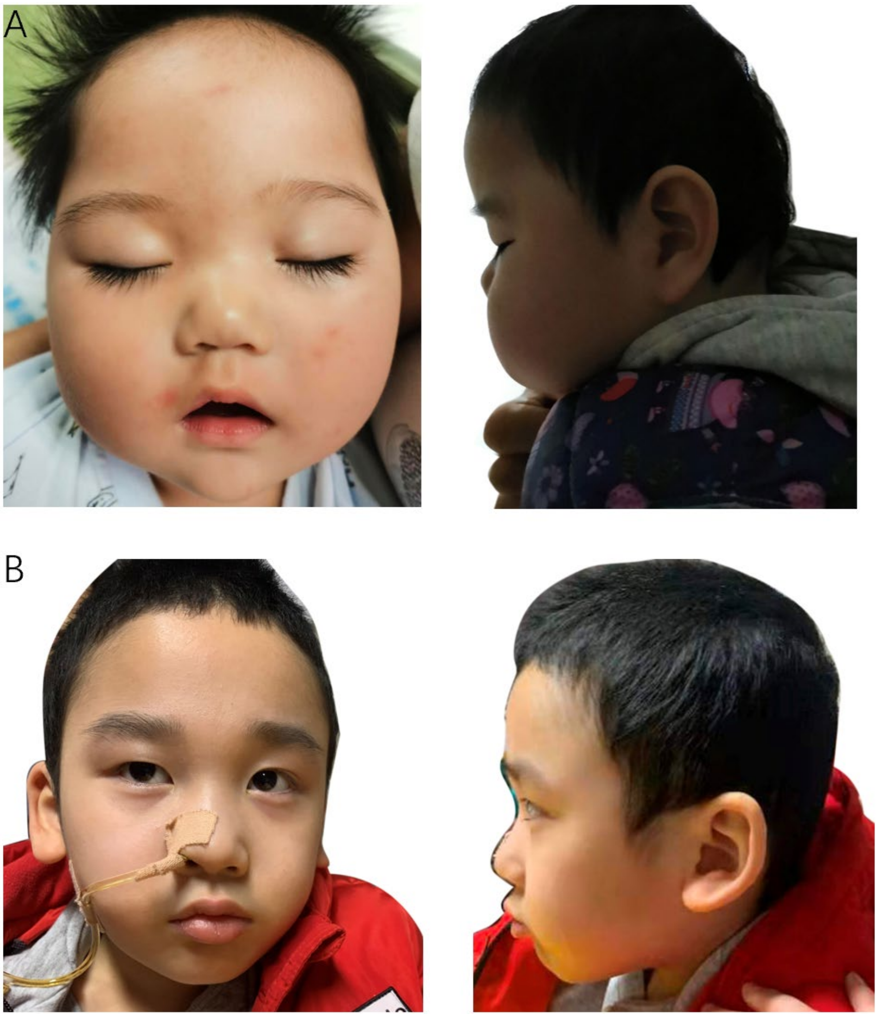

Supplementary Figure 1. Photos of Individuals with KMT2E Variants. (A) Case 1, 5 months old with microcephaly, slightly large foreheads, prominent cheeks, and long eyelashest; (B) Case 2, 5 years old with no obvious distinct facial features.

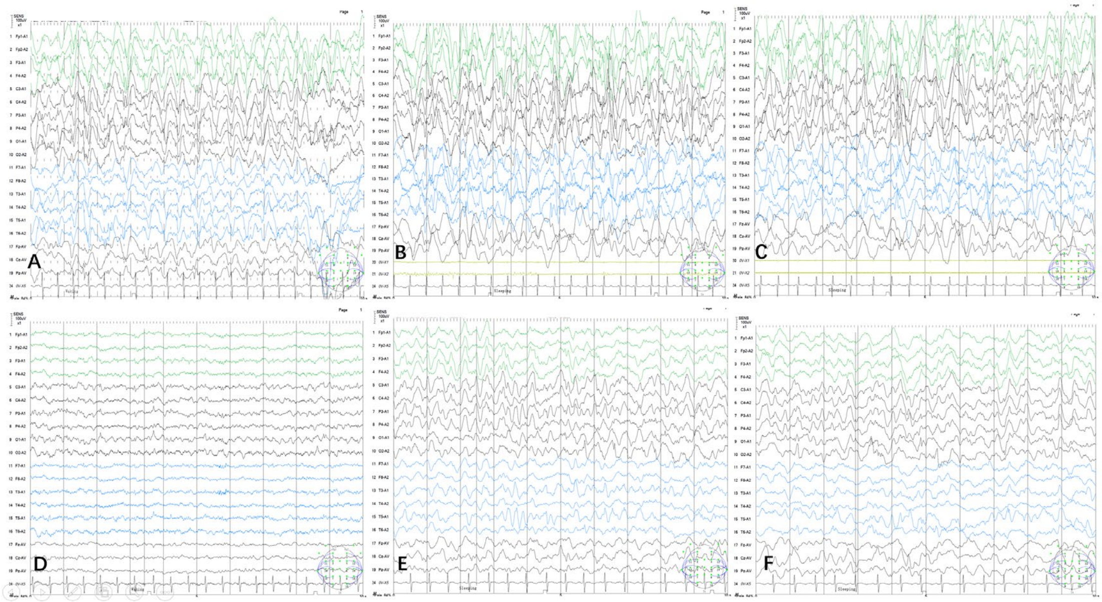

Supplementary Figure 2. EEG of Case 1. EEG (referential montage) on admission demonstrated high amplitude bilateral widespread asynchronous 3-4Hz slow activities intermingled with spikes, maximal over left posterior temporal region during awake (A) and sleep(B, C). After treatment, background EEG showed low to moderate amplitude 4-5Hz slow activities over the occipital region with occasional small sharp waves over left posterior quadrant(D). EEG during sleep showed moderate amplitude widespread sharply contoured slow waves, prominent in the occipital region (E, F). [SENS *10 uv/mm, HF *70 Hz, TC *0.3 s, CAL*50 Hz]

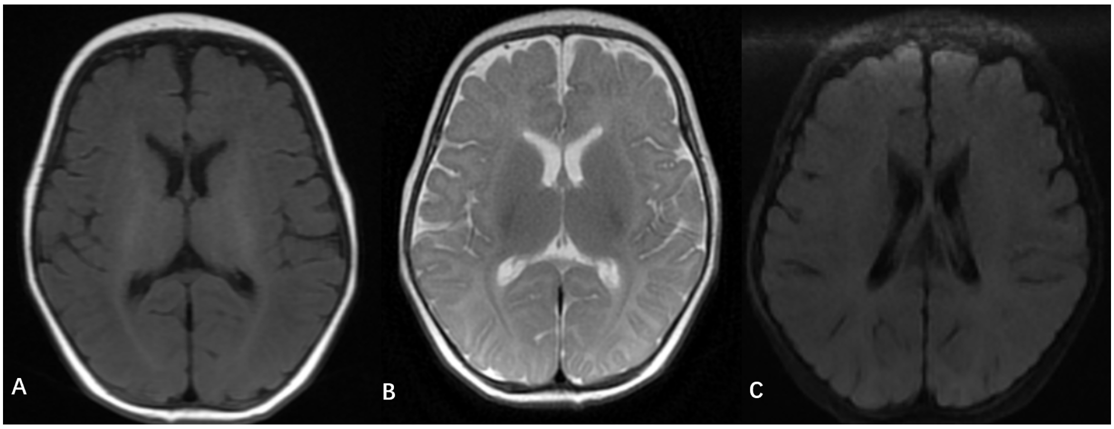

Supplementary Figure 3. Magnetic resonance imaging (MRI) of Case 1. Axial T1-weighted (A), T2-weighted (B), and fluid-attenuated inversion recovery (C) sequences showed no abnormal signals.

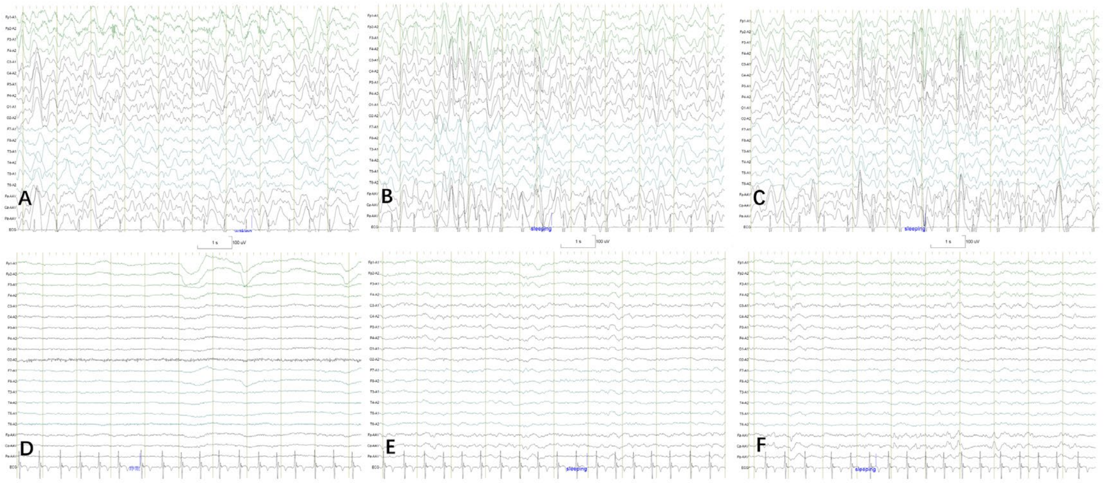

Supplementary Figure 4. EEG of Case 2. On admission, the EEG showed diffuse 3-4Hz slow activities with sharp and spike waves over the frontal, temporal, and parietal regions during awake (A,) and sleep (B, C). EEG on the last follow up showed generalized low amplitude wave without clear sleep staging (D, E, and F). [SENS *10 uv/mm, HF *70 Hz, TC *0.3 s, CAL*50 Hz]

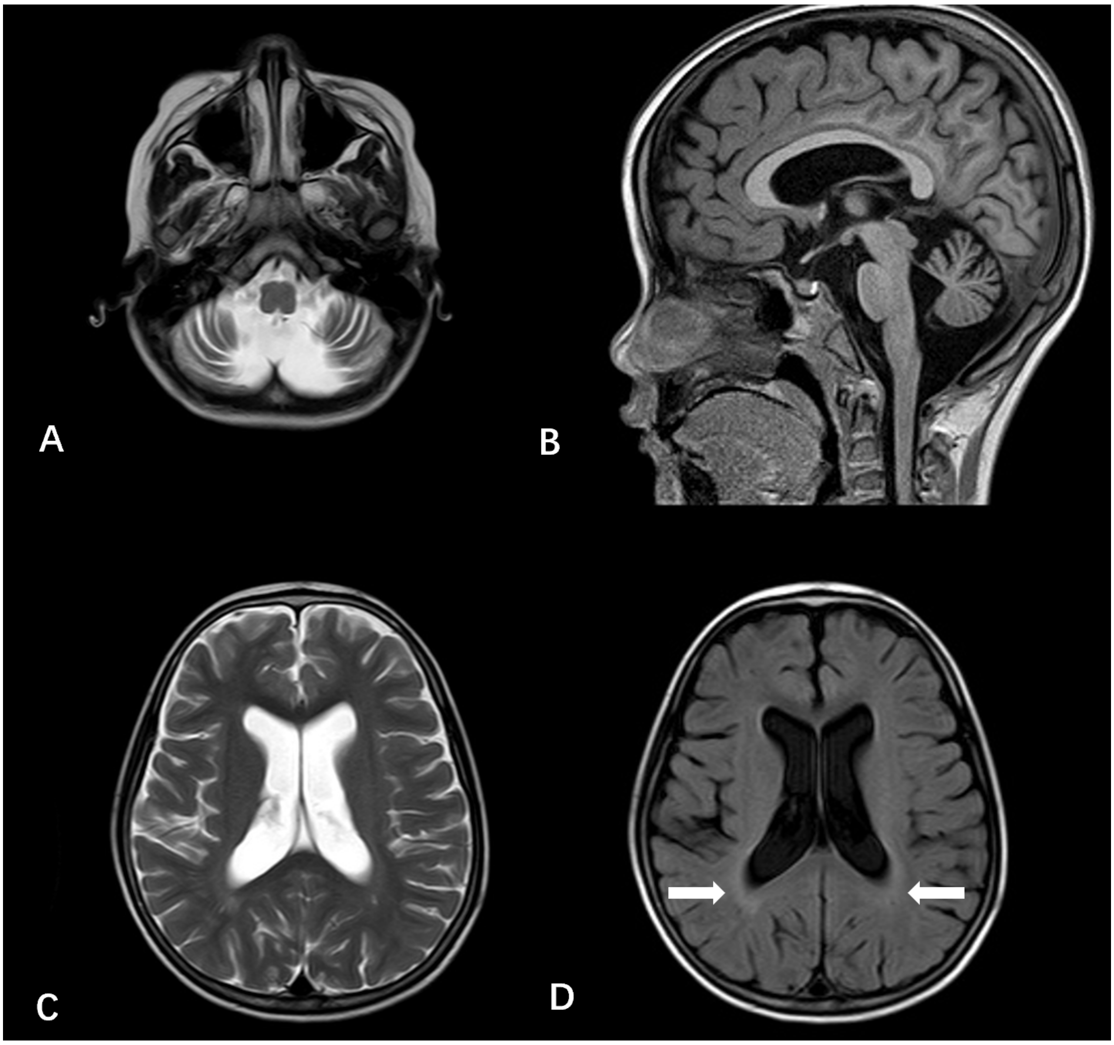

Supplementary Figure 5. Magnetic resonance imaging (MRI) of the patients (case 2). Axial T2-weighted (A, C), sagittal T1-weighted (B) and fluid-attenuated inversion recovery (D) sequences showed cerebellum atrophy, as well as broadening of lateral and third ventricle. subtle bilateral and symmetrical hyperintense signal changes in peritrigonal white matter.
